# Leptin-Independent Association Between SNVs in the Leptin Gene and HDL-C and Apo-AI in Children

**DOI:** 10.3390/ijms262411906

**Published:** 2025-12-10

**Authors:** Olga Pomares, Iris Pérez-Nadador, Francisco Javier Mejorado-Molano, Alejandro Parra-Rodríguez, Leandro Soriano-Guillén, Carmen Garcés

**Affiliations:** 1 Lipid Research Laboratory, Instituto de Investigación Sanitaria Fundación Jiménez Díaz (IIS-FJD, UAM), 28040 Madrid, Spain; olga.pomares@iis-fjd.es (O.P.); iris.perezn@iis-fjd.es (I.P.-N.); 2 Department of Pediatrics, Instituto de Investigación Sanitaria Fundación Jiménez Díaz (IIS-FJD, UAM), 28040 Madrid, Spain; fmejorado@quironsalud.es (F.J.M.-M.); alejandro.parra@quironsalud.es (A.P.-R.); lsoriano@fjd.es (L.S.-G.)

**Keywords:** leptin, *LEP* SNVs, lipids, prepubertal children

## Abstract

Polymorphisms in the leptin gene (*LEP*) have been associated with leptin levels and anthropometric variables; however, their association with lipid profiles remains under study. We aimed to determine the relationship between *LEP* single-nucleotide variants (SNVs) and body mass index (BMI), leptin levels, and lipid profiles in prepubertal children. This cross-sectional study included a population-based sample of 1270 males and females aged 6-to-8 years. Lipid and leptin levels were quantified, and the SNVs G19A and G2548A were analyzed by real-time PCR using predesigned TaqMan™ Genotyping Assays. We found that both *LEP* SNVs were significantly associated with leptin levels after adjusting for sex. No significant associations between the studied SNVs and BMI were observed in our population. Additionally, both SNVs were associated with apolipoprotein AI (Apo-AI) levels in females, whereas G2548A was also associated with high-density lipoprotein cholesterol (HDL-C) levels after adjusting for sex. These associations remained statistically significant after adjusting for leptin levels. No association was found between SNVs and other lipid variable levels. Our results indicate that polymorphisms in the *LEP* gene influence not only leptin levels but also lipid metabolism, as evidenced by their association with Apo-AI and HDL-C, independent of plasma leptin concentrations.

## 1. Introduction

Leptin is an adipocyte-derived hormone that, in addition to maintaining energy homeostasis, plays an important role in regulating lipid metabolism. It promotes lipolysis and fatty acid oxidation and downregulates lipogenesis [1,2]. In this regard, besides being analyzed for its association with obesity, the association of leptin levels with lipid profiles has been extensively studied in adults [3,4,5] and children [6,7,8,9], describing an association with an adverse lipid profile. We described a significant positive association of leptin with triglyceride levels and a negative association with high-density lipoprotein cholesterol (HDL-C) and apolipoprotein AI (Apo-AI) concentrations in a cohort of prepubertal children [10]. The association we have described between plasma leptin levels and non-esterified fatty acid (NEFA) concentrations in this cohort of children aged 6–8 years is particularly interesting [11].

Two single-nucleotide variants (SNVs) in the leptin gene, G19A and G2548A, located in the 5′ untranslated region (UTR) of the human leptin gene, have been reported to be associated with body mass index (BMI) and obesity, as well as leptin levels, but with discordant results [12,13,14,15,16,17,18,19,20]. Studies in children also focused on these SNVs are scarce [21,22,23,24]. We previously described a sex-dependent relationship of G2548A with leptin levels and obesity in a population-based sample of Spanish adolescents [25].

In contrast, the association of these SNVs with lipid levels has been less explored, and the results of the analysis to date have been inconclusive [26,27,28,29]. Although the role of leptin in lipolysis is well known [1,2], to our knowledge, its association with NEFA levels has not been investigated.

In our study, we analyzed whether the SNVs G19A and G2548A in the leptin gene are associated with BMI, leptin levels, and lipid profile, particularly NEFA, in early life by analyzing a large sample population of prepubertal children.

## 2. Results

Table 1 summarizes the age, BMI, leptin levels, and lipid parameters of the study population according to gender. The mean age was the same for males and females (7.2 ± 0.6 years), and the mean BMI value did not significantly differ between genders (Table 1). However, compared to males, females exhibited significantly higher mean levels of leptin, triglycerides (TGs), low-density lipoprotein cholesterol (LDL-C), and apolipoprotein B (Apo-B), and lower mean levels of Apo-AI (Table 1).

### 2.1. Genotype and Allele Frequencies of LEP SNVs

The characterization and genotype distributions of the analyzed *LEP* SNVs are presented in Table 2. The minor allele frequencies (MAFs) of the G19A and G2548A polymorphisms in the leptin gene were 34.0% and 43.0%, respectively. The observed allelic distributions were consistent with Hardy–Weinberg equilibrium (HWE) principles.

For both *LEP* SNVs (G19A and G2548A), GG denotes homozygosity for the major ancestral allele (G), GA indicates heterozygosity for G and A alleles, and AA represents homozygosity for the minor allele (A).

### 2.2. Association Between LEP SNVs and BMI and Leptin Levels

The association between leptin SNVs G19A and G2548A and BMI and leptin levels was assessed using a univariate ANOVA adjusted for sex (Table 3). No statistically significant differences in BMI were observed for either *LEP* SNVs. However, the association between *LEP* SNVs and leptin levels showed significant differences across the genotypes. For the *LEP* SNV G19A, a progressive increase in leptin levels was observed in the GG, GA, and AA genotypes (Table 3), with AA carriers showing significantly higher leptin levels than GG carriers (*p* = 0.033). For the G2548A *LEP* SNV, we observed the opposite effect; the presence of the A allele was significantly associated with lower leptin levels, with GG carriers showing significantly higher leptin levels than A allele carriers (*p* = 0.016) (Table 3).

### 2.3. Association Between LEP SNVs and Lipid Parameters

**Figure 1:**
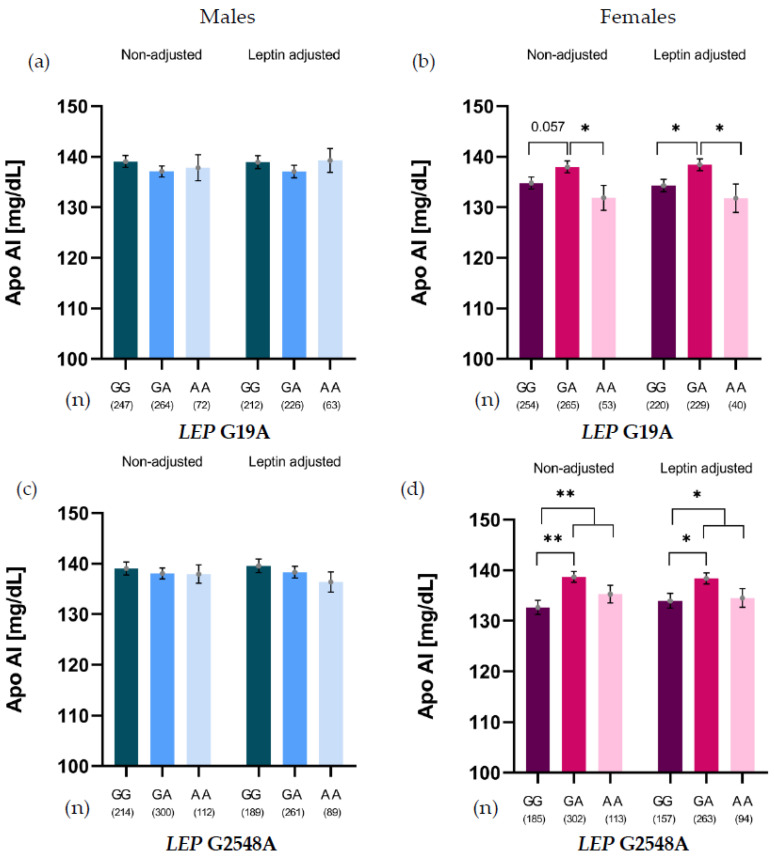
Apo-AI levels of *LEP* G19A genotypes in males (**a**) and females (**b**), and *LEP* G2548A genotypes in males (**c**) and females (**d**), non-adjusted and adjusted for leptin levels. * *p* < 0.05; ** *p* < 0.01.

The relationship between the *LEP* SNVs G19A and G2548A and lipid variables was analyzed by stratifying the results by sex. A significant association between both SNVs and Apo-AI levels was only observed in females (Figure 1).

For G19A, heterozygotes (GA) showed higher Apo-AI levels than GG carriers (*p* = 0.057) and AA homozygotes (*p* = 0.035). This association was leptin-independent, as the difference persisted after adjusting for leptin, revealing significant differences between GG and GA carriers (*p* = 0.012) and between AA and GA carriers (*p* = 0.025) (Figure 1b). For G2548A, GA carriers showed significantly higher Apo-AI levels than GG carriers. This association persisted even after adjusting for leptin levels (Figure 1d).

**Figure 2:**
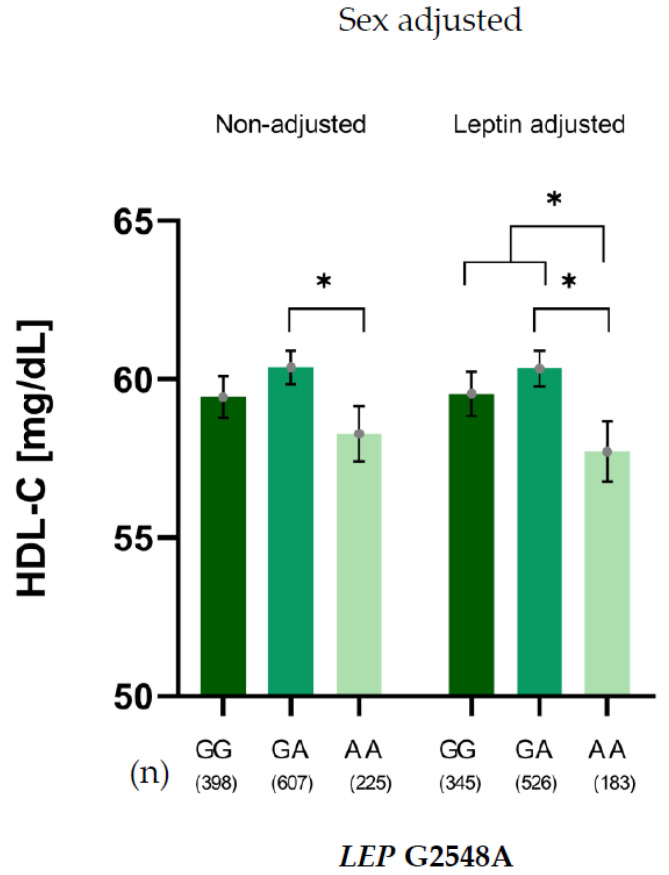
HDL-C levels of *LEP* G2548A genotypes adjusted for sex, non-adjusted and adjusted for leptin levels. * *p* < 0.05.

A similar pattern was observed for the relationship between the G2548 *LEP* SNV and HDL-C levels, adjusted for sex. This relationship persisted after further adjustment for leptin levels, confirming that the association between the G2548A genotype and HDL-C levels was independent of leptin. In this context, G allele carriers showed significantly higher HDL-C levels than AA carriers (Figure 2).

We examined the association between *LEP* SNVs genotypes and other lipid variables (total cholesterol (TC), TG, LDL-C, Apo-B, and NEFA levels). No statistically significant differences were observed across genotypes for any of the lipid parameters.

## 3. Discussion

Given the significant role of leptin in regulating energy homeostasis, the influence of polymorphisms in the leptin gene on obesity and leptin plasma levels has been extensively analyzed in adults [12,13,14,15,16,17,18,19,20] and children [21,22,23,24,25]. However, their association with lipid metabolism remains unclear [26,27,28,29]. In our study of prepubertal children, the *LEP* SNVs G19A and G2548A were not associated with BMI but were significantly associated with leptin levels. These variants were chosen because they are the most widely studied SNVs in the leptin gene and have been central to previous research investigating leptin regulation and metabolic outcomes.

The association of these SNVs in the leptin gene with anthropometric variables and obesity has been reported in several studies in children, including samples of different ages, with discordant results among studies [21,22,23,24,25]. In this sense, we have previously analyzed a cohort of older children (adolescents aged 12-to-16 years) describing a significantly lower presence of the minor allele (A) for the G2548A *LEP* SNV in overweight/obese females than in normal-weight females. We found that BMI was significantly lower in AA carriers than in GG female carriers [25], supporting the existence of a sex-dependent association. The different associations described in the literature and in our own studies depending on sex and age may be due to the different age- and sex-related hormonal status of the studied populations and point out that the lack of association with BMI in our cohort of younger children could be related to the status of sexual development.

Our study showed that both SNVs were related to leptin concentrations in females. For G19A, GG carriers showed lower leptin levels than A-allele carriers. For G2548A, the GA and AA genotypes had significantly lower plasma leptin levels than GG carriers. Previously, in our cohort of adolescents, we described an association between the G2548A polymorphism and plasma leptin levels, with carriers of the A allele having lower leptin levels [25]. Other studies in children have also reported an association between the AA genotype and lower leptin levels [14,21,22,23].

Functional activity data concerning the activity of *LEP* polymorphisms remains controversial. As they are located within regions involved in gene regulation, these *LEP* SNVs do not directly alter the amino acid sequence or structure of the leptin protein. Instead, it is possible that they might affect gene transcription, mRNA stability, and translation [22]. Concerning the G2548A *LEP* SNV, Hoffstedt et al. [30] reported that A allele carriers showed higher mRNA *LEP* levels compared to GA/GG carriers, postulating that this promoter variant likely increases transcription by modifying transcription factors binding affinity, enhancing *LEP* promoter activity, influencing gene expression, and leading to altered circulating leptin levels. Additional studies, such as the study from Kolic et al. [31], found that A allele carriers had significantly higher *LEP* mRNA levels compared to GG carriers in a cohort of patients with relapsing-remitting multiple sclerosis; however, Song et al. [29], associated the A allele with reduced *LEP* mRNA expression. Together, these inconsistencies indicate that the regulatory consequences of the G2548A variant remain incompletely understood.

In this sense, an examination of the influence of the rs1137101 variant (*LEPR*) on *LEP* mRNA levels across various tissues revealed a significant negative association with *LEP* mRNA in cultured fibroblasts and the tibial artery, yet a significant positive correlation in tissues such as subcutaneous adipose, brain, and thyroid [29]. We speculate that the regulatory impact of the G2548A variant, and potentially other *LEP* SNVs, on gene expression may be tissue specific. Although this specific example involves a different variant, it powerfully illustrates the differential regulatory landscape of the *LEP* locus across distinct human tissues.

The relevant finding in our study is that, when analyzing the association of *LEP* SNVs with lipid concentrations, we found a relationship with HDL-C and Apo-AI concentrations that remained significant after adjusting for leptin levels. These results strongly suggest that the identified polymorphisms influence lipid metabolism independent of their potential effect on leptin expression or secretion, indicating a direct or pleiotropic mechanism that avoids systemic leptin concentration.

The relationship between circulating leptin and plasma lipid levels remains unclear. Some studies in children have observed a negative association between leptin levels and HDL-C, which has not been observed in other studies or has been described as a positive or negative association depending on sex [6,7,8,9]. We previously described a significant negative association between plasma HDL-C and Apo-AI levels in this cohort of prepubertal children [10]. Thus, polymorphisms that affect leptin levels may be associated with these parameters. However, in our study, we observed that leptin gene polymorphisms were associated with lipid levels independent of leptin levels, distinguishing the genetic effect from the endocrine axis.

Leptin exerts its function by binding to and activating the leptin receptor (LEPR), mainly in the hypothalamus [32,33]. However, the presence of functional leptin receptors in white adipose tissue suggests a direct action of leptin on adipocyte metabolism [34]. Leptin participates in lipid metabolism by inhibiting lipogenesis and stimulating lipolysis in adipose tissue [1,2]. In the liver, a central player in systemic lipid metabolism, leptin can inhibit the gene expression of key lipogenic enzymes, such as acetyl-CoA carboxylase and fatty acid synthase, resulting in decreased de novo lipid synthesis [35]. Furthermore, there is evidence suggesting that leptin can inhibit adipocyte proliferation [36] and exert autocrine and paracrine lipolytic effects in white adipose tissue, both in vitro and ex vivo [37,38].

The leptin concentration-independent association observed in our study suggests that *LEP* variants may influence lipid metabolism through alternative mechanisms. These SNVs may be in linkage disequilibrium with regulatory elements affecting *LEPR* splicing or transcript stability, particularly in hepatocytes and adipocytes [29,30]. Another plausible mechanism is that *LEP* variants may modulate hepatic HDL-C clearance pathways, suggesting that they might indirectly affect the downstream transcription factors that regulate the activity of the main HDL-C receptor, Scavenger Receptor B1 (SR-B1) [39,40]. This could alter HDL-C catabolism and Apo-AI incorporation into lipoproteins without affecting systemic leptin levels. Collectively, these findings indicate that *LEP* genetic variation may subtly impact lipid metabolism and reverse cholesterol transport through tissue-specific or regulatory mechanisms independent of circulating leptin.

The strength of our study lies in the fact that we analyzed a homogeneous and broad sample of Caucasian 6-to-8-year-old children without disparity in age and genetic background, as well as free of hormonal influence acting as a confounding factor in our analysis of the relationship between the SNVs and the parameters under study. A limitation of our study is the lack of information on the functional activity of the SNVs which would help clarify their association with the studied variables. An additional important limitation of our study is the lack of physical activity and socioeconomic factors that may affect the variables under study.

In conclusion, the present study suggests that polymorphisms within the leptin gene exert associations independent of leptin concentrations, affecting lipid concentrations. This is substantiated by the observed associations with HDL-C and Apo-AI levels, independent of circulating plasma leptin concentrations. Given the central role of HDL-C and Apo-AI in cardiovascular protection, such variants may contribute to interindividual susceptibility to dyslipidemia and long-term cardiovascular risks.

## 4. Materials and Methods

### 4.1. Subjects

The sample population included 1270 prepubertal children (638 males and 632 females), aged 6–8 years old, who participated in a cross-sectional study examining cardiovascular risk factors in Spain [41]. All participants were confirmed to be free of pre-existing endocrine, metabolic, hepatic, or renal disorders.

The study was presented orally to the School Board of each participating school. Subsequently, a letter outlining the study goals and procedures was sent to the parents of all children invited to participate in the study. The parents were required to provide written consent for their children to participate in the study. The study protocol complied with the Helsinki Declaration guidelines and Spanish legal provisions governing human clinical research. The study was approved by the Clinical Research Ethics Committee of the Fundación Jiménez Díaz (approval reference number: PIC105-2023 FJD, 15 September 2023).

### 4.2. Anthropometric Measurements

Measurements were taken with the children wearing light clothing and barefoot. Weight and height were recorded to the nearest 0.1 kg and 0.1 cm, respectively, using a standardized electronic digital scale and portable stadiometer, respectively. These values were subsequently used to calculate BMI, expressed as weight in kilograms divided by the square of the height in meters (kg/m^2^).

### 4.3. Biochemical and Genetic Determinations

*Sample collection and processing*: Fasting (12 h) venous blood samples were obtained early in the morning by venipuncture. Blood was drawn into two types of Vacutainer tubes: one containing EDTA-Na2 as an anticoagulant and the other containing a serum gel separator. Following immediate centrifugation, the resulting fractions (plasma and serum) were separated and immediately stored at −70 °C to ensure preservation for subsequent biochemical and genetic analyses.

*Biochemical assays:* TC and TG levels were determined enzymatically using a Technicon RA-1000 Autoanalyzer (Menarini Diagnostics, Naples, Italy). HDL-C was measured using an RA-1000 analyzer after precipitation of Apo-B-containing lipoproteins with phosphotungstic acid and Mg^2+^ (Boehringer, Mannheim, Germany). LDL-C was calculated using the Friedewald formula. Plasma Apo-AI and Apo-B concentrations were quantified using immunoephelometry (Dade Behring, Marburg, Germany). The intra-assay coefficients of variation (CVs) for the main analytes were as follows: cholesterol, 1.4%; TG, 1.7%; Apo-AI, 1.6%; and Apo-B, 4.8%.

NEFA levels were measured using the Wako NEFA-C kit (Wako Industries, Osaka, Japan). Leptin levels were measured by ELISA with a commercial kit (Leptin EIA-2395; DRG, Marburg, Germany).

*LEP SNVs genotyping*: Genomic DNA (gDNA) was isolated from leukocytes according to standard procedures. The quantity and quality of the recovered gDNA were assessed by UV-spectrophotometry using a NanoDrop spectrophotometer (ND-1000, Thermo Fisher Scientific).

The selected *LEP* SNVs, G19A (rs2167270) and G2548A (rs7799039), were genotyped by Real-Time PCR, using predesigned TaqMan™ SNV Genotyping Assays from Thermo Fisher Scientific (Waltham, MA, USA); (C__15966471_20 and C__1328079_10, respectively). The QuantStudio3^®^ Real-Time PCR System (Applied Biosystems, Thermo Fisher Scientific) was used for allelic discrimination. qPCR was performed using a mixture containing 10 ng genomic DNA and TaqMan™ Master Mix (Thermo Fisher Scientific). The samples were cycled under the following conditions: 95 °C for 10 min, 95 °C for 15 s, and 60 °C for 1 min, repeated over 40 cycles.

### 4.4. Statistical Analysis

Statistical analyses were performed using SPSS software package (version 25.0; IBM, New York, NY, USA) and GraphPad Prism version 8 statistical software (San Diego, CA, USA). Descriptive statistics are presented as mean ± standard deviation (SD).

The normality of all continuous variables was assessed using the Kolmogorov–Smirnov test. Variables with skewed distributions were logarithmically transformed prior to the statistical analysis. Initial differences in means between males and females were tested using Student’s *t*-test. Analysis of variance (ANOVA) was used to compare quantitative variables across genotypes for males and females separately. Post hoc comparisons between genotype groups were conducted using Tukey’s test whenever statistically significant differences were detected (*p* < 0.05). Univariate analyses were used to compare lipid variables across *LEP* genotypes, adjusting for both leptin levels and sex.

## Figures and Tables

**Table 1:** Age, BMI, and biochemical parameters of the study sample population by sex. Values are expressed as mean ± SD.

	6-to-8-Year-Olds		*p*-Value
	Males (n = 638)	Females (n = 632)	
**Age** (years)	7.2 ± 0.6	7.2 ± 0.6	ns
**BMI** (kg/m^2^)	16.9 ± 2.4	17.0 ± 2.5	ns
**Leptin** (ng/mL)	4.1 ± 5.1	6.1 ± 6.4	***
**TC** (mg/dL)	181.8 ± 26.2	183.6 ± 28.4	ns
**TG** (mg/dL)	71.2 ± 25.4	74.2 ± 26.5	*
**LDL-C** (mg/dL)	107.3 ± 25.4	110.2 ± 26.9	*
**Apo-B** (mg/dL)	68.9 ± 14.1	71.4 ± 14.9	**
**HDL-C** (mg/dL)	60.2 ± 13.1	58.8 ± 13.3	ns
**Apo-AI** (mg/dL)	138.3 ± 19.1	135.6 ± 18.9	*
**NEFA** (mmol/L)	0.7 ± 0.3	0.7 ± 0.3	ns

* *p* < 0.05, ** *p* < 0.010, *** *p* < 0.001.

**Table 2:** Description, genotype, and allele distribution of the studied SNVs in the *LEP* gene.

*LEP* SNVs ^a^	Genotype	% (n)	Allele (%)	HWE *p*-Value
**G19A** (rs2167270)	GG	43.5 (508)	G (0.66)	0.3811
g.128241296 G>A	GA	45.8 (534)	A (0.34)	
5′UTR	AA	10.7 (125)		
**G2548A** (rs7799039)	GG	32.4 (402)	G (0.57)	0.8240
g.128238730 G>A	GA	49.3 (612)	A (0.43)	
5′UTR	AA	18.3 (227)		

*LEP*, chr. 7 (7q31.1); Locus NC_000007.14. HWE: Hardy–Weinberg equilibrium. ^a^ Human reference genome GRCh38 (Hg38), genome browse. software (https://www.ncbi.nlm.nih.gov/).

**Table 3:** BMI and leptin levels by *LEP* SNVs adjusted for sex.

BMI (kg/m^2^)				
***LEP* G19A**	GG (n = 508)	GA (n = 534)	AA (n = 125)	*p*-Value
	16.92 ± 0.13	16.99 ± 0.12	17.03 ± 0.23	ns
***LEP* G2548A**	GG (n = 402)	GA (n = 612)	AA (n = 227)	
	17.10 ± 0.15	16.93 ± 0.11	16.86 ± 0.19	ns
**Leptin (ng/mL)**				
***LEP* G19A**	GG (n = 508)	GA (n = 534)	AA (n = 125)	GG vs. GA+AA (0.063) GG vs. AA (0.033)
	4.83 ± 0.26	5.21 ± 0.27	6.02 ± 0.57	
***LEP* G2548A**	GG (n = 402)	GA (n = 612)	AA (n = 227)	GG vs. GA+AA (0.017) GG vs. AA (0.016)
	5.67 ± 0.30	4.93 ± 0.25	4.36 ± 0.42	

*p*-value: Tukey (ANOVA, post hoc test).

## Data Availability

The datasets analyzed during the current study are available from the corresponding author upon reasonable request and with permission from the Jiménez Díaz Foundation Clinical Research Ethics Committee.

## Abbreviations

The following abbreviations are used in this manuscript:

Apo-AI	Apolipoprotein-AI
Apo-B	Apolipoprotein-B
BMI	Body Mass Index
HWE	Hardy–Weinberg Equilibrium
HDL-C	High-density lipoprotein cholesterol
LDL-C	Low-density lipoprotein cholesterol
MAF	Minor Allele Frequency
NEFA	Non-Esterified Fatty Acid
SR-B1	Scavenger Receptor B1
SNV	Single-Nucleotide Variant
TC	Total Cholesterol
TG	Triglyceride
UTR	Untranslated region
